# Editorial: Targeting Neuroinflammation in Central Nervous System Disorders: Uncovering Mechanisms, Pharmacological Targets, and Neuropharmaceutical Developments

**DOI:** 10.3389/fphar.2021.771610

**Published:** 2021-12-01

**Authors:** Mariela Fernanda Pérez, Flavia Saravia, María Graciela Castro, Claudia Bregonzio

**Affiliations:** ^1^ Instituto de Farmacología Experimental Córdoba (IFEC-CONICET), Departamento de Farmacología, Facultad de Ciencias Químicas, Universidad Nacional de Córdoba, Córdoba, Argentina; ^2^ Facultad de Ciencias Exactas y Naturales, Universidad de Buenos Aires, Cordoba, Argentina; ^3^ Department of Neurosurgery, School of Medicine, University of Michigan, Ann Arbor, MI, United States

**Keywords:** neuroinflammation, Alzheimer´s disease, drug abuse and addiction, traumatic brain injury, autophagic dysfunction, lymphatic drainage and uptake

In this special issue we present a series of reviews and research papers written from leading authors highlighting neuroinflammation as a common target to overcome and understand brain disorders. Neuroinflammation plays a key role in Parkinson, Alzheimer, schizophrenia, major depression and drug addiction, among others. Some threads include: autophagic dysfunction (essential for the removal of unnecessary cellular constituents and dysfunctional components), dysfunctional central nervous system (CNS) lymphatic drainage (dampened cleansing of molecules prone to aggregation and reduced immune cell egress into the draining cervical lymphatic nodes), traumatic brain injury and psychostimulants and alcohol abuse disorders. The growing evidence shows that attenuation of neuroinflammation brings beneficial effects against neurodegeneration and cognitive deficits associated with a plethora of CNS disorders. Considering this last issue, the identification of its most relevant biological processes and possible pharmacological targets remains a major challenge.

Keeping in mind the role of neuroinflammation in psychiatric disorders and its implications in mediating worsening of the symptoms, it is important to highlight the search of biomarkers for early diagnosis and improved treatment. In this sense it is important to consider a recent study performed in patients with major psychiatric disorders showing alterations in cerebrospinal fluid (CSF) inflammatory cytokine levels. Hidese et al. found that CSF interferon-β levels, among 19 cytokines tested was significantly higher in patients with schizophrenia, or bipolar disorder when compared to healthy controls. This represents novel evidence showing prominent statistical differences between psychiatric groups and healthy controls. Along the same lines, in a very interesting computational exploration of the molecular network associated to neuroinflammation in Alzheimer’s disease (AD), Idrissi et al. found that 94 proteins were significantly associated. Over the scientific literature they identified eleven key proteins with the highest ability to control neuroinflammatory processes significantly associated with AD and pharmacological compounds with single or pleiotropic actions acting on them. These results could help in the prioritization of diagnostic and target-engagement biomarkers as well as in the development of therapeutic strategies against neuroinflammation in AD.

The autophagic process is implicated in the removal of unnecessary or dysfunctional proteins and damaged organelles *via* the lysosomal machinery. For this reason, it is critical for the maintenance of neuronal function and to provide neuroprotection in neurodegenerative disorders. Adequate regulation of autophagy is associated with beneficial outcomes in neurodegenerative diseases (Nixon, 2013; Cho et al., 2020). The interplay between autophagy and inflammation is complex, although neuroinflammation is implicated in autophagic dysfunction. In this sense, it is known that upregulation of autophagy promotes microglial polarization toward the M2 phenotype by the suppression of M1 markers (Jin et al., 2018; Cho et al., 2020). In this respect, Lee et al. found that dimethyl fumarate reduced NO production and the expression levels of genes associated with the M1 phenotype, including TNF-α and IL-6 and phagocytosis in microglia, both associated with the M2 phenotype. These data suggest that this compound leads to the induction of autophagy in microglia and its anti-inflammatory effects are partially mediated through an autophagy-dependent pathway.

It is well known that following brain injury or in neurodegenerative diseases, astrocytes become reactive and may suffer pathological remodeling, they lose their homeostatic functions facilitating neurodegeneration by maintenance of the pro-inflammatory environment. A deeper understanding of the cellular and molecular mechanisms involved in the astroglial response and neuroinflammation are needed to develop pharmacological interventions that will lead to novel therapeutic strategies. In this regard, Villarreal et al. showed that pathological neuroinflammatory conversion of reactive astrocytes is induced by microglia and involves chromatin remodeling. These results open a new perspective in pharmacological interventions that affect astroglial pathological remodeling and point out to epigenetic changes as a potential pharmacological target to interfere with pathological astroglial phenotype stabilization.

Neuroinflammation is also a risk factor for neurodegenerative disease such as AD. Vinuesa et al. compiled data supporting the role of inflammation and insulin resistance as risk factors for AD and explored potential therapeutic targets. Considering that there has been a global rise of type II diabetes and obesity prevalence, it becomes necessary to understand how changes in metabolic function can lead to an increased risk for premature brain aging and the development of neurodegenerative disorders such as AD. In this respect, the interplay between inflammation and insulin resistance could represent a potential therapeutic target to prevent or ameliorate neurodegeneration and cognitive impairment.

The morphology and unique functional features of meningeal lymphatics have a close relation with several brain disorders. Recent studies have tested the effects of boosting the function of the meningeal lymphatics in mouse models of AD. Since a pathological hallmark of AD is the accumulation of extracellular amyloid plaques rich in aggregated amyloid beta (Aβ) peptides (Ittner and Gotz, 2011) impairment in Aβ clearance could contribute to its accumulation (Wisniewski and Goni, 2014). Moreover, the presence of intracellular neurofibrillary tangles is another pathological hallmark of this neurodegenerative disease, which is composed by hyperphosphorylated forms of the microtubule-associated protein Tau (Ittner and Gotz, 2011). Pereira das Neves et al. highlighted and exhaustively described the evidence supporting the notion that an impaired meningeal lymphatic drainage in AD could promote both Aβ and Tau accumulation in the brain, affecting disease severity and aggravating cognitive decline. Induction of traumatic brain injury (TBI) in mice resulted in a substantial decrease in meningeal lymphatic drainage as early as 2 hours post-injury, which was only fully restored 2 months later (Bolte et al., 2020). In accordance, CSF flow was altered in TBI (Johanson et al., 2011), and intracranial pressure was markedly increased 2 hours post-injury (Bolte et al., 2020). These data suggest that there is an early temporal window right after brain injury, suitable for pharmacological interventions, in order to prevent secondary injury mechanisms and reduce the development of long-term disabilities, including cognitive, affective and physical impairments, as well as neurodegenerative pathologies. Regrettably, none of the pharmacological agents used in the clinic to manage the TBI sequelae can inhibit the neuroinflammatory cascade (Carney et al., 2017). Montivero et al. proposed brain insulin-like growth factor 1 (IGF-1) over-expression, considering IGF-1 neuroprotective and anti-inflammatory effects (Zheng et al., 2000; Carlson and Saatman 2018; Serhan et al., 2019). This treatment, performed as early as 15 min after TBI, was effective not only in reducing oxidative-stress markers, but also in improving the cognitive deficits observed long-term after mild TBI in adult rats.

In a very interesting review, Namba et al. describe the neuroimmune mechanisms as novel treatment targets for substance abuse disorders and associated comorbidities. This report presents recent studies analyzing the neurobiology of substance abuse that have exposed a significant role in neuroimmune signaling as a mechanism for drugs of abuse changes in synaptic plasticity and contribute to drug abuse-related behaviors. In the same lines, Xu et al. showed in a study performed in female mice, that alcohol consumption increased the expression of neuroinflammation markers. Accordingly, Villavicencio-Tejo et al. found that fenofibrate (peroxisome proliferator-activated receptor alpha agonist) administered during ethanol withdrawal blunted ethanol-induced astrogliosis and restores the levels of glutamate transporter in ethanol-administered adolescent rats. Moreover, the activation of PPARα by fibrates inhibits neuroinflammation, in models other than ethanol consumption. In relation to other drug of abuse, Basmadjian et al. showed that D-amphetamine is able to induce oxidative stress, transient angiogenesis, and long-lasting astroglial and microglial reactivity in the prefrontal cortex. These effects were prevented with an angiotensin II AT1 receptor blocker, candesartan. To this respect, it was shown that AT1-R blockade exerts protective effects over gliosis and pro-inflammatory compounds released in several animal models of neuroinflammation.

Taken together all findings presented in the Research Topic, point out that neuroinflammation is a common denominator between diverse CNS-associated pathologies such as neurodegenerative diseases, acute injuries, metabolic, psychiatric and drug abuse induced disorders. The ability to modulate neuroinflammation could provide a novel therapeutic opportunity to improve the outcomes in these devasting conditions.
